# A Critical Review of Alcohol Reduction Methods for Red Wines From the Perspective of Phenolic Compositions

**DOI:** 10.1002/fsn3.70401

**Published:** 2025-06-08

**Authors:** Jingxian An, Zhipeng Zhang, Anwen Jin, Muqiu Tan, Shilong Jiang, Yilin Li

**Affiliations:** ^1^ Chemical and Materials Engineering The University of Auckland New Zealand; ^2^ Jiangxi Copper Technology Institute co., Ltd. Nanchang China; ^3^ Heilongjiang Feihe Dairy co., Ltd Beijing China

**Keywords:** alcohol reduction, dealcoholized wine, phenolic compositions, phenolic loss, sensory attributes, wine quality

## Abstract

The growing demand for low‐alcohol and nonalcoholic wines has sparked significant interest in alcohol reduction methods within the wine industry. This review examines various techniques for reducing alcohol content in red wines, focusing on their impact on phenolic compositions, which are crucial for wine quality and health benefits. Alcohol reduction methods have been analyzed across viticultural processes, fermentation techniques, and postfermentation treatments including membrane separation, vacuum distillation, and supercritical extraction. Main findings indicate that while all alcohol reduction methods can lead to some degree of phenolic loss, certain techniques like osmotic distillation show promise in preserving phenolic compositions. The review explores strategies to compensate for phenolic loss through mechanical methods, blending techniques, and food additive additions. Notably, the loss of certain phenolic compositions, particularly volatile phenols associated with smoke taints and small‐molecular‐weight phenolics, can sometimes positively influence sensory attributes. The paper concludes by highlighting the need for a comprehensive sensory attributes database for dealcoholized wines to assist in selecting optimal methods for balancing sensory attributes and wine quality. Further research is needed to explore how available methods can improve the sensory attributes of dealcoholized wines.

## Graphical abstract

Alcohol reduction methods in red wines impact phenolic compositions and sensory attributes. Osmotic distillation shows promise in preserving phenolic compounds during dealcoholizing. Mechanical and blending methods can compensate for phenolic loss in dealcoholized wines.

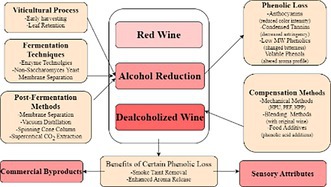

## 1 Introduction

Red wine plays an important role in the human diet around the world due to its comparably high phenolic composition compared to white wines and beers. Phenolic composition not only contributes to red wines' sensory attributes like high color intensity, moderate astringency, and balanced tastes, which can provide consumers with pleasure and enjoyment and have positive relationships with wine quality (An et al. 2023). In addition, phenolic composition is also beneficial for scavenging free radicals, enhancing fat oxidation, reducing stress, inflammation, DNA damage, aging, and cancer, and improving the immune system and cardiac functioning (Akyereko et al. 2021).

However, alcohol in red wines is associated with a range of health and social risks, such as liver diseases and cardiovascular problems. These risks led the World Health Organization to publish a global strategy to curb harmful alcohol use in 2010. In addition, winemakers in some countries must pay taxes if the ethanol content exceeds 14.5% v/v (Catarino and Mendes 2011). In response to these concerns, the market for low‐alcohol wines (alcohol content with 0.5%–1.2% v/v), reduced‐alcohol wine (alcohol content with 1.2%–6.5% v/v), and nonalcoholic wines (alcohol‐free or dealcoholized wines) has grown significantly in recent years (Akyereko et al. 2021; Day et al. 2024). For example, low‐alcohol wine consumption constitutes about 40% of total wine consumption in the USA (Akyereko et al. 2021). France experienced a 25% increase in new no‐alcohol consumers (Day et al. 2024). In addition, alcohol consumption has been declining in Australia over the last 15 years, mostly driven by younger generations who are reducing or eliminating their alcohol intake to improve their health and for fear of addiction (Shaw et al. 2023).

**FIGURE 1:**
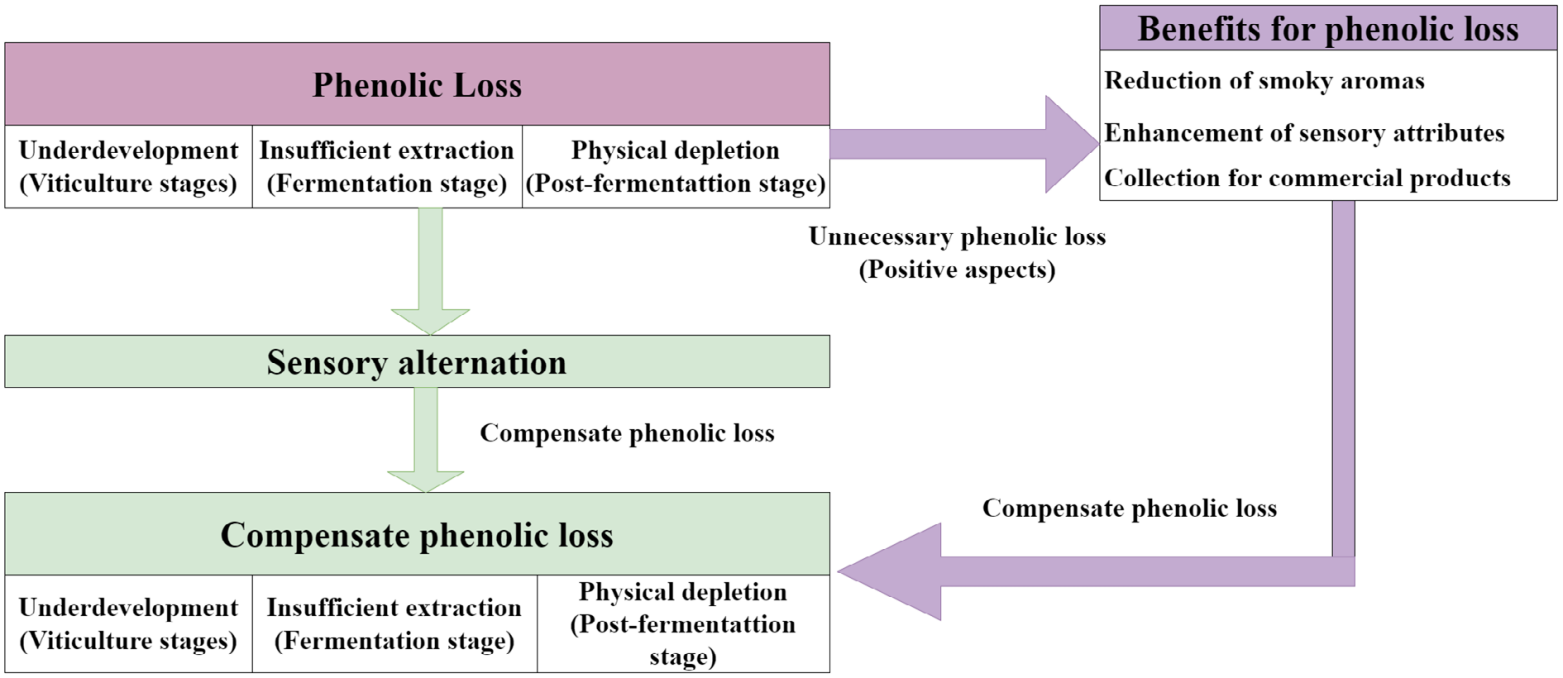
The influence of alcohol reduction methods on phenolic compositions in red wines.

To reduce alcoholic content, it is essential to understand how alcohol is formed in alcoholic beverages. Alcohol is a primary metabolite produced by yeasts during the alcoholic fermentation of grape sugars present in must. To reduce or remove the alcohol content, many methods have been developed, which include viticultural methods like leaf removal, fermentation methods such as adding enzymes, postfermentation methods like blending, separation methods, thermal processes, mechanical assistance, and other processes (Day et al. 2024). Regardless of the method used, phenolic compositions experience varying degrees of loss (as shown in Figure 1). Phenolics are a group of compounds that contribute to the color, taste, and mouthfeel of wines, and their presence is crucial for the overall quality of the product. Currently, researchers are primarily focused on exploring various methods for removing alcohol content and summarizing their applications. However, improperly removing alcohol content will create barriers for consumers who are resistant to purchasing low‐alcohol wines (Shaw et al. 2023). It is common sense that phenolic compositions have significant roles in determining red wines' tastes.

The aim of this review is to analyze the advantages and disadvantages of various alcohol reduction methods and their impact on phenolic composition in red wines, with the goal of guiding winemakers in selecting appropriate techniques that meet consumer demands for dealcoholized wines. Additionally, it will explore how phenolic compositions influence the sensory attributes of dealcoholized red wines. This review will also discuss methods to compensate for phenolic loss, including mechanical techniques, blending methods, and the use of food additives. It will examine whether these phenolic compensation methods can positively influence sensory attributes. Furthermore, the paper will explore potential positive effects of phenolic loss on the sensory profile of the final product.

## 2 Phenolic Loss in Viticulture Stages

### 2.1 Viticultural Processes

Viticultural processes, including earlier harvesting and leaf retention, are two methods to maintain lower sugar levels in grapes, which indicate that alcohol content can be reduced rather than totally removed. However, grapes harvested earlier may lack phenolic maturity, leading to a lower phenolic concentration. Meanwhile, when grapes are phenolic immature, the skin phenolic compounds are not easily extracted, even when present at high concentration, yet high concentrations of the astringent seed tannins can be present (Pérez‐Porras et al. 2021). By contrast, grape anthocyanin and skin tannin concentrations increased as ripening progressed, which can increase monomeric anthocyanin and wine condensed tannin (polymerized flavanols) concentrations in the corresponding wine (Bindon et al. 2013). Phenolic compositions are an important parameter for grape and wine quality (Gutiérrez‐Escobar et al. 2021; Wang et al. 2021).

In contrast, leaf removal is a common operation during viticultural processes to increase total anthocyanins and phenolic concentration, as shown in a previous study on *Vitis vinifera* L. cv. Graciano and Carignan grapevines (Tardaguila et al. 2010). Similarly to Osrečak et al.'s (Osrečak et al. 2016) finding, leaf retention can contribute to hydroxycinnamate content and malvidin‐3‐glucoside biosynthesis. However, removing leaves can also increase grape sugars, leading to higher alcohol content in the resulting wine. Therefore, viticultural processes alone, such as earlier harvesting and leaf retention, are not the best options to control alcohol content in wines.

### 2.2 Phenolic Loss in Fermentation Stage

Fermentation is a crucial stage in the production of alcoholic beverages, particularly for red wines, which includes crushing, fermentation, and maturation. During fermentation, yeast is added to the grape must, which is crushed grape juice containing seeds, skins, and stems. The yeast consumes the sugars in the must and converts them into alcohol and carbon dioxide. There are three main methods used to reduce alcohol content during the fermentation stage: enzyme technologies, low alcohol‐producing yeast strains, and membrane separation techniques.

Enzyme technologies and low alcohol‐producing yeast strains can directly reduce the alcohol content in the final product. Enzyme technologies work by breaking down the sugars in the must into smaller, nonfermentable sugars, which the yeast cannot convert into alcohol. Low alcohol‐producing yeast strains such as non‐Saccharomyces yeast, on the other hand, are genetically modified or selectively bred to produce less alcohol during fermentation. In addition to these methods, membrane separation techniques can be applied to remove the residual sugar, which can otherwise be converted into alcohol (García‐Martín et al. 2010). This technique involves passing the must through a semipermeable membrane that allows the sugar molecules to pass through while retaining the other components of the must. Nanofiltration is the most common method used to remove sugar in grape juices, which is consistent with summaries from El Rayess et al. (2024) and García‐Martín et al. (2010).

These methods can both reduce alcohol content. However, during the maceration phase of red winemaking, where the grape solids remain in contact with the juice, phenolic compounds are extracted by alcohol. For example, anthocyanins are extracted from skins, and condensed tannins are extracted from skins and seeds (Setford et al. 2017). It seems that reducing alcohol content at this stage with a single technique would negatively affect the extraction of all phenolic compositions.

### 2.3 Phenolic Loss in Postfermentation Stage

Apart from lowering the concentration of phenolic compositions, both viticultural methods and fermentation stages cannot completely remove the alcohol content. In this situation, other postfermentation alcohol reduction techniques have been used to remove alcohol content, including membrane separation, vacuum distillation, spinning cone distillation, and supercritical extraction method.

With the removal of alcohol content and some water loss, although some phenolic compounds are lost, the concentration of phenolic compositions in wines may increase. The following section will analyze the influence of diverse methods on phenolic compositions.

#### 2.3.1 Membrane Separation

In the wine industry, membrane separation typically includes microfiltration (MF), ultrafiltration (UF), nanofiltration (NF), and reverse osmosis (RO). These are pressure‐driven membrane operations, usually classified according to their pore sizes and driving forces (Charcosset 2021; Conidi et al. 2012). The pore size is inversely correlated with the required driving force: the smaller the pore size, the higher the driving pressure needed. Specifically:

- MF, with a pore size of 0.2 μm, is used to filter wines and remove solids and microorganisms (El Rayess et al. 2024).
- UF has a pore size range of 0.001 to 0.1 μm and can retain species with molecular weights (referred to as molecular weight cut‐off) between 300 and 106 Da (Charcosset 2021).
- NF, with pore sizes of 0.001–0.005 μm, retains solutes with molar masses between 1000 and 3000 Da (Charcosset 2021).
- RO has the smallest pore size (< 0.001 μm) and retains solutes with molar masses below 1000 Da (Charcosset 2021). RO has unique properties and challenges:

1. It is permeable in both alcohol and water.
2. After filtration, water must be added back to the dealcoholized wine (El Rayess et al. 2024; García‐Martín et al. 2010).
3. This creates legal issues in countries where water addition is prohibited (García‐Martín et al. 2010).

Other membrane processes, such as osmotic distillation (OD) and pervaporation (PV), are also used in membrane separation to remove volatile compounds and ethanol (El Rayess et al. 2024). Unlike pressure‐driven methods, these are driven by vapor pressure differences. The volatile compounds collected during these processes can potentially be treated with absorbents to remove volatile phenols. The remaining aromatic fractions could then be added back to smoky taint‐affected red wines.

During membrane separation, the phenolic composition of wine can be altered. The influence of different membrane types on dealcoholized wines with diverse phenolic compositions has been summarized in Table 1.

**TABLE 1:** The phenolic composition changes in dealcoholized wines.

Wine Type	Membrane process	Initial alcohol content (% v/v)	Final alcohol content (% v/v)	Academic findings
Aglianico red wine (Liguori et al. 2013)	OD	—	Up to 2% reduction	No significant differences between original and dealcoholized wine.
Cabernet Sauvignon (Gil et al. 2013)	RO	14.8%	13.8%, 12.8%	No significant changes in total polyphenols, proanthocyanins, and diverse anthocyanins after alcohol reduction.
Cabernet Sauvignon (El Rayess et al. 2024)	NF	—	—	The concentration of tannins decreased by 4.8% and 2.4%, respectively, and total 10% reduction in the total polyphenolic index.
cv. Montepulciano d'Abruzzo (Corona et al. 2019)	OD	13.2%	Reductions of 4.9%, 6.3%, 7.8%, 9.2% and 10.5%	No significant differences for flavonoids and phenolic compounds in all dealcoholized samples.
Kékfrankos and Kékoportó (Banvolgyi et al. 2016)	NF	—	4%–6% (after reconstitution)	Total anthocyanins increased by 2.5–3 times in the wine concentrates.
cv. Montepulciano d'Abruzzo (Russo et al. 2019)	RO followed by OD	13.2%	Reductions of about 5%, 6% and 8%	Significantly decreased in total anthocyanins after RO, but the amount remained unchanged after two OD cycles. The concentration of total phenols increased due to permeation of both ethanol and water during RO. Total phenolic content remained constant in the subsequent OD cycles.
Five different types of red wines (Pham et al. 2019)	RO‐evaporative perstraction	—	Reductions of 0.5% to 5%	Dealcoholized wines were slightly more concentrated than pretreatment wines, resulting in increased anthocyanins and phenolic compounds.
Cabernet Sauvignon, Merlot, and Tempranillo (Bogianchini et al. 2011)	RO	12.7%	4% (with further reduction to 2% by adding 25 L water	Anthocyanins and phenolic acids' concentrations were not significantly changed.

When using membrane processes with porous membranes, membrane separation has very low selectivity toward low‐molecular‐weight solutes and volatile compounds. Consequently, some low‐molecular‐weight phenolic compositions, such as catechin and phenolic acids, are lost during membrane separation by passing across the membrane and are contained in the permeates (as shown in Table S1). Meanwhile, smoky phenols usually have a low molecular weight of less than 200 Da, which will not be retained in the majority of membrane separations (as shown in Supplementary Table 2). Additionally, some phenolic compositions aggregated with polyphenols have a strong affinity to the polar membranes, leading to severe fouling, while other phenolic compositions may be absorbed by the membrane (Cassano et al. 2011). These factors can lead to changes in total anthocyanins, total flavonoids, and total phenolics.

Currently, NF and RO are the most popular membrane‐based processes used for beverage dealcoholizing (Catarino and Mendes 2011). However, based on Table 1, it can be concluded that osmotic distillation (OD) is the most comparable available method to preserve phenolic composition in red wines. OD can prevent the entry of the aqueous solution into the pores, while the use of water as a stripper creates an ethanol vapor pressure difference between both membrane sides. This increases the ethanol flux and reduces the water activity across the membrane, thereby limiting its transport (Esteras‐Saz et al. 2023). Notably, when the alcohol content has been reduced to within 2% v/v, the phenolic composition shows insignificant changes among red wines.

#### 2.3.2 Vacuum Distillation and Spinning Cone Column (SSC)

Distillation, using either evaporators or distillation columns, is the most common thermal‐based method for removing alcohol from wine. However, the process of dealcoholizing requires heating and evaporation of 50%–70% of the wine to reduce the alcohol content to below 0.5% v/v. This method is not perfect due to two main reasons. Firstly, it can cause the loss of wine during thermal processes (Jadhav et al. 2021). Secondly, thermal treatments may result in the deterioration of nutritional and sensory attributes of wines (Jadhav et al. 2021).

To address these problems, vacuum distillation apparatus has replaced distilling vessels to remove more ethanol at much lower temperatures (Pickering 2000). For example, in the production of nonalcoholic wines, the pressure inside the plant is reduced to between 0.05 and 2 bar, enabling distillation to occur within a temperature range from 20°C to 30°C. This low‐temperature distillation can help prevent negative changes in the wine's aroma components (Schulz et al. 2024). Motta et al. (2017) found that when wine's alcohol content is 5% (v/v), the concentration of anthocyanins and polyphenols in dealcoholized red wines treated by membrane separation is similar to original wines. However, the dealcoholized wines treated by vacuum distillation have significantly higher concentrations of anthocyanins and polyphenols.

The spinning cone column (SCC) vacuum distillation is a special form of vacuum distillation that consists of vertical cones rotating at high speeds. The feed is introduced at the top of the column into the first spinning cone. SCC vacuum distillation operates at high speeds and low temperatures and is regarded as a highly effective and cost‐effective method in the food industry for retaining and conserving volatile aroma compounds from liquids such as fruit juices, tea, and coffee (Belisario‐Sánchez et al. 2009). In the wine industry, SCC vacuum distillation has been used to produce grape must concentrate, remove sulfur dioxide from grape must, recover volatile aroma compounds, and reduce ethanol concentrations in wines (Sam et al. 2021).

Although SCC vacuum distillation is performed at mild operation temperatures (26°C–35°C), the operation takes place in two steps. Firstly, the volatile aromatics are separated at a low temperature (approx. 28°C). Secondly, alcohol separation occurs at a higher temperature (approx. 38°C) (Schulz et al. 2024). After ethanol separation, the aromatic fraction is added back to the wine, resulting in a long and expensive operation (Diban et al. 2008). Currently, when red wine has been dealcoholized to an alcohol content of less than 0.3% (v/v), the concentrations of total phenolics, flavanols, anthocyanins, resveratrol, gallic acid, epicatechin, catechin, caffeic acid, ρ‐coumaric acid, rutin, myricetin, quercetin, and malvidin have increased or decreased only slightly, indicating that SCC has minimal impact on wine phenolic compositions (Belisario‐Sánchez et al. 2009). By contrast, in smoke‐tainted red wines, including Shiraz Sangiovese and Petit Verdot Sangiovese, total anthocyanins, tannins, and phenolics increased somewhat, while volatile phenols were also removed (Puglisi et al. 2022).

#### 2.3.3 Supercritical Extraction Method

Traditional methods such as membrane separation and vacuum distillation have the disadvantage of eliminating aromas together with ethanol. Nowadays, supercritical fluid extraction, particularly using supercritical CO_2_ technology, is a promising technique. There are two different countercurrent extraction steps employed to produce a low‐ethanol content wine. In the first step, the extraction and recovery of aroma from the original wine are the targets, while in the second step, the extraction is driven toward dealcoholizing the aroma‐free product (obtained in the first step) to an ethanol content lower than 1 wt% (Ruiz‐Rodríguez et al. 2012).

CO_2_ is a nonpolar molecule, which makes it more efficient at extracting nonpolar compounds and less efficient for extracting polar molecules (e.g., phenolic compositions), potentially minimizing phenolic loss (Ghafoor et al. 2012). This is consistent with findings that nonpolar molecules easily migrate into the fluid phase when CO_2_ is used as the supercritical solvent (de Andrade Lima et al. 2021). Some low‐molecular‐weight and volatile phenolic compositions may be lost during the process, particularly when increasing temperature and decreasing pressure allow CO_2_ to transition from fluid to gas. However, this typically does not result in significant overall phenolic loss. Moreover, the extracted alcohol can serve as a valuable byproduct, increasing its industrial utility.

Based on Section 2, we can infer that phenolic loss traits at different stages are as follows:

In the viticulture and fermentation stages:

- Anthocyanins and condensed tannins will be lost.

In the postfermentation stage:

- Small‐molecular‐weight phenolic compositions will be lost during membrane separation.
- Volatile phenols will be lost during vacuum distillation.
- Limited volatile phenols will be lost in the SCC vacuum distillation.
- Supercritical CO_2_ extraction can remove some nonpolar and limited small polar phenols.

## 3 Sensory Alterations

As discussed in Section 2, it can be inferred that certain phenolic compounds are lost during the dealcoholizing process. This section will analyze the influence of specific phenolic compositions on various sensory attributes.

### 3.1 Astringency and Bitterness

Astringency is not only a taste but also a feeling of dryness or roughness that results from increased friction between the tongue and the surfaces inside the mouth (Landon et al. 2008). When astringency is excessive, wines are considered “aggressive” and/or “rough”. By contrast, when the mouthfeel is less astringent, the wines will taste “flat”, “insipid” and “uninteresting” (González‐Muñoz et al. 2022). As opposed to astringency, bitterness is unpleasant, but it does give a wanted sensory attribute for red wines (Soares et al. 2013).

Proanthocyanidins, also called condensed tannins, are compounds responsible for bitterness and astringency in wine. Skin tannins, which combine with polysaccharides and proteins, contribute to softness and roundness but can impart herbaceous notes if the fruit is not fully ripe. Seed tannins give structure to the wine but can also impart excessive astringency (Ivanova et al. 2012). Additionally, condensed tannins, which occur as galloylated species either conjugated with anthocyanins or in free form, are largely responsible for red wine astringency (Ivanova et al. 2012). During both the viticultural and fermentation stages, the loss of condensed tannins and anthocyanins can negatively affect red wines' astringency and bitterness. These findings are consistent with Renata et al. (2016), who found that wines made from more mature grapes have higher concentrations of tannins, which are positively associated with astringency. Furthermore, Pham et al. (2020) reported that underdeveloped phenolic compositions could lead to green or herbaceous characters and negative color characteristics.

Regarding membrane separation, research has shown that low‐molecular‐weight phenolic compounds are easily lost during the process. Previous studies have found that low‐molecular‐weight flavan‐3‐ols, such as catechins and epicatechin, are responsible for bitterness in wine. Epicatechin is more bitter than its stereoisomer catechin, and both of these compounds are more bitter than procyanidin trimers (Soares et al. 2017). Phenolic acids with small molecular weights can also significantly contribute to astringency, bitterness, and sourness (Duizer and Langfried 2016). The loss of phenolic acids will cause unbalanced tastes in red wines.

### 3.2 Color

The color of red wine is one of the first features perceived by consumers and can greatly influence its commercial acceptance (He et al. 2012). Anthocyanins are the main pigments in red wines that determine their color, and their concentrations are influenced by grape variety, traditional maceration, and storage conditions (González‐Neves et al. 2016). During both the viticultural and fermentation stages, the loss of anthocyanins can negatively affect the color of red wines. In contrast, dealcoholized wines produced by vacuum distillation have been found to retain high concentrations of total anthocyanins, suggesting that these dealcoholized red wines may have a deeper red color compared to the original wines. Wines dealcoholized by membrane separation or spinning cone column show only slight differences in red color compared to the original red wines.

### 3.3 Aromas

In the postfermentation stage, volatile phenols are easily lost during membrane separation, vacuum distillation, and spinning cone column processes, which can alter the aromas of dealcoholized red wines. Previous research has found that volatile phenols are negatively associated with wine quality (Sáenz‐Navajas et al. 2015). The presence of volatile phenols is mostly regarded as off‐flavors that mask the natural fruity notes of wine, even at concentrations below the olfactory threshold (Binati et al. 2020). Therefore, it can be hypothesized that removing volatile phenols through the aforementioned methods may not only reduce alcohol content but also potentially improve the aromas of red wines.

In summary, it can be inferred that viticultural methods, fermentation techniques, membrane separation, and vacuum distillation can significantly affect the astringency, bitterness, color, and aroma of dealcoholized red wines—crucial factors in determining wine quality. The impact of membrane separation and vacuum distillation on wine quality, whether positive or negative, depends on individual situations. The spinning cone column method can positively influence the aromas of dealcoholized red wines, while supercritical extraction methods have limited effects on phenolic compositions.

## 4 Compensate Phenolic Loss in Red Wines

According to Sections 2 and 3, it can be inferred that phenolic loss can alter the sensory attributes of dealcoholized red wines, particularly due to the loss of condensed tannins, anthocyanins, and total phenolics. These changes might negatively affect red wines' commercial value and overall quality (Kassara and Kennedy 2011). To address these issues, diverse methods including mechanical techniques, blending approaches, and the addition of food additives are employed (Table 2). In fact, different methods have different advantages and disadvantages, which will affect the overall sensory quality of the wine as well as the production cost.

**TABLE 2:** Strategies applied to compensate phenolic loss in red wine.

Method	Processing techniques	Strength	Limitation
Mechanical Methods	Nonthermal treatments: High‐power ultrasound (HPU), Pulsed electric fields (PEF), High‐pressure processing (HPP)	‐Reduces alcohols and enhances polyphenol extraction. ‐ Solves membrane fouling issues and avoids phenolic loss during postfermentation.	‐ Operational parameters need to be precisely controlled to prevent increased alcohol concentration. ‐ Requires specialized equipment and technical expertise.
Blending Methods	Mixing grape juice with water, dealcoholized wine or prefermentative juice	‐ Lowers alcohol content without significantly altering important quality components. ‐ Cost‐effective, environmentally friendly, and safer compared to other alcohol reduction methods.	‐ Mixing ratios may vary for different grape varieties. ‐ May have impacts on volatile compounds.
Food Additive Additions	Addition of phenolic acids, byproducts extracts, or mannoproteins.	‐ Compensates for phenolic loss and improves fruity and floral aromas. ‐ Utilizes byproducts (grape seeds and skins) to Enhance phenolic content.	‐ Consumer acceptance of certain additives may be low, especially for less knowledgeable consumers. ‐ Need to ensure the safety and legality of additives.

### 4.1 Mechanical Methods

Mechanical methods are mainly used to recover more phenolic compounds to compensate for the shortcomings of phenolic underdevelopment in the viticulture stages, insufficient extraction in the fermentation stage, and phenolic loss in the postfermentation stage.

Compared to thermal treatments like thermovinification, flash release, or microwaves, which may affect the quality and stability of phenolic compositions (Pérez‐Porras et al. 2021), nonthermal treatments are preferred for treating early harvested grapes. Early harvested grapes contain less sugar, which can guarantee a lower alcohol content in wines after fermentation. However, these grapes also have insufficient bioactive compositions like phenolic compositions and anthocyanins. To address this issue, nonthermal treatments such as high‐power ultrasound (HPU), pulsed electric fields (PEF), and high‐pressure processing (HPP) can be used to increase phenolic compositions in early harvested grapes during brewing (Pérez‐Porras et al. 2021; Zhang et al. 2020). For example, in a study by Martínez‐Pérez et al. (2020) and Pérez‐Porras et al. (2021), early harvested grapes treated with HPU after crushing produced wines with characteristics very similar to those made with more mature grapes, especially regarding total phenol and tannin content. These wines also achieved the highest scores in aroma and mouthfeel quality descriptors in a sensory analysis, despite having an alcohol content 15% lower than the latter. PEF could permit attaining the highest phenolic content, anthocyanin content, and tannin content in wines (El Darra et al. 2013). Meanwhile, HPP can increase total phenolic content in Syrah and Pinot Noir wines with 600 MPa (Van Wyk et al. 2021).

In addition to dealing with phenolic underdevelopment, mechanical methods can be used to extract more phenolic compositions during fermentation. Ultrasound is an effective technique to reduce higher alcohols in wines and can effectively enhance the extraction of polyphenols, including anthocyanins and tannins, from grape seeds and skins during fermentation (El Darra et al. 2013). For example, the use of PEF allows, through electroporation, the permeabilization of cell tissues, facilitating the extraction of phenolic compositions (Pérez‐Porras et al. 2021). HPU, which generally comprises frequencies between 20 and 40 kHz with an energy level high enough to produce acoustic cavitation, has shown promising results in extracting phenolic compositions from grapes to optimize the maceration process. Usually, high tannin extraction occurs at 20 kHz, whereas anthocyanin extraction is favored at 28 kHz (Martínez‐Pérez et al. 2020).

During the postfermentation stage, when alcohol content in red wines is separated using techniques such as membrane separation, it is easy to encounter membrane fouling due to polyphenols and polysaccharides present in red wines (Vernhet and Moutounet 2002). This fouling can hinder the separation process and increase the temperature during membrane separation, which can cause the hydrolysis of some heat‐sensitive phenolic compositions. Meanwhile, phenolic compositions will be absorbed in the membrane due to the aggregation of wine constituents at the pore entrance on the membrane surface (Vernhet and Moutounet 2002). Water hammer generated by mechanical methods like ultrasound can effectively solve membrane fouling and avoid phenolic loss at the postfermentation stage (Aslam et al. 2022).

However, the study carried out by Pérez‐Porras et al. indicated that when using HPU at 20 kHz, the alcohol content might rise along with the increase in the tannin level (Pérez‐Porras et al. 2021). Consequently, operational parameters are necessary to prevent the alcohol concentration from increasing. Moreover, suitable equipment and technical expertise are needed.

### 4.2 Blending Methods

Blending wines is a very commonly used but not very well‐studied area in winemaking. Prefermentative juice substitution with early harvest wine has the potential to produce lower alcohol wines without critically modifying color, total tannins, total anthocyanins, or total phenolics. This method only marginally changes volatile compounds, total polymeric pigments, and sensory profiles (Fanzone et al. 2020; Longo et al. 2018; Renata et al. 2016; Schelezki et al. 2020). On the other hand, certain volatile compounds such as volatile acid might be influenced by substitution with early harvest wine (Schelezki et al. 2020). In addition, several studies have investigated the production of red wine using the addition of either water or low alcohol wine to substitute a proportion of juice, hence decreasing wine alcohol levels without greatly “diluting” important wine quality components such as anthocyanins and tannins or sensory characteristics as well (Montevecchi et al. 2024; Schelezki et al. 2020; Teng et al. 2020). Moreover, blending the original wine with appropriately dealcoholized samples has proven to be an effective strategy for preserving the bouquet and color of reduced‐alcohol wines. This approach can broaden the consumer base by catering to people who prefer low‐alcohol options but still wish to savor wines with superior quality and complexity rather than a flat taste (Montevecchi et al. 2024; Silva 2024).

The blending method is frequently utilized to enhance wine quality through the mixture of various types of wines. When grape juice is mixed with early harvest wine or water, this approach can lower the alcohol content without the need for additional machinery or additives during wine production. Thus, blending methods are safer, more environmentally friendly, and more cost‐effective compared to other alcohol reduction methods. Nevertheless, the mixing ratios may differ for each grape type (Longo et al. 2018; Schelezki et al. 2020). Different mixing ratios to balance alcohol content and sensory properties are still required (Montevecchi et al. 2024).

### 4.3 Food Additive Additions

Based on Section 2, it can be inferred that phenolic composition in the viticulture and fermentation stages can be negatively influenced, and phenolic composition in postfermentation can cause some loss as well. Along with phenolic loss, aroma will evaporate as well. To make up for this problem, phenolic acid addition is one alternative, which can not only compensate for phenolic loss but also improve fruit and floral aromas. For example, phenolic acid addition (gallic acid or ρ‐coumaric acid) in grape juice along with terpene glycosides during alcoholic fermentation can prolong terpene release by inhibiting terpene glycoside hydrolysis and free terpene volatilization (Wang et al. 2021).

During wine production, byproducts such as grape seeds and skins still contain a considerable quantity of phenolic substances. Phenols can be extracted from these byproducts and added to wine (Trošt et al. 2016). Additionally, seeds can be added prior to fermentation (Rivero et al. 2017). Mannoprotein is a common additive at the postfermentation stage for protecting wine color. While adding mannoprotein before fermentation can significantly increase the concentrations of anthocyanins, tannins, and phenolic acids in red wine (Yue et al. 2021).

However, compared to nonthermal treatment and blending methods, food additives can be partially accepted by consumers. For example, consumers considered natural flavorings and colors, and additives associated with health benefits (e.g., vitamins, minerals, and omega‐3 fatty acids), to be acceptable food additives, irrespective of their level of wine knowledge (Saltman et al. 2015). In contrast, the use of winemaking additives, even commonly used and legally permitted additives, such as tartaric acid, preservatives, oak chips, and tannins, was considered far less acceptable, particularly by less knowledgeable consumers (Saltman et al. 2015).

## 5 The Benefits of Certain Phenolic Loss

Phenolic loss can sometimes cause an imbalance in sensory attributes due to ethanol evaporation or minimal water removal. However, in certain cases, phenolic loss may play a positive role by removing smoky aromas and enhancing sensory attributes.

### 5.1 Reduction of Smoky Aromas

According to a previous study, it has been found that volatile phenols and their glycoconjugates are also associated with smoke taints. The removal of smoke‐derived volatile phenols can be used to ameliorate smoke taint in wine. For example, compounds such as 2,6‐dimethoxyphenol, 4‐ethylguaiacol, thymol, guaiacol, and carvacrol are potentially associated with a smoky aroma, and their removal can reduce smoky aromas (Wang and Chambers IV 2018). Guaiacol, 4‐methylguaiacol, o‐cresol, m‐cresol, p‐cresol, guaiacol β‐D‐glucoside, and m‐cresol β‐D‐glucoside could contribute to smoky aromas but have molecular weights lower than 300 Da (Parker et al. 2012). Additionally, nonsmoky phenolic compounds can also contribute to smoked flavor when combined with either a smoky phenolic compound or another nonsmoky phenolic compound (Wang and Chambers IV 2018).

The feasible method to address this issue involves first collecting aromatic fractions and removing volatile phenols from them, then adding the aromatic fractions without volatile phenols back to the grape juice. There are many available methods that can be used to remove volatile phenols. Usually, volatile phenols have small molecular weights and contribute to smoky taints. In detail, during this process, the aromatic fraction is passed through an ion exchange column to remove volatile phenols via solid‐phase adsorption during the SCC process. After this treatment, the purified aromatic fraction is blended with the juice before fermentation begins (Puglisi et al. 2022). Alternatively, in pervaporation, where substances pass through the membrane and change from liquid to vapor, most of the aroma compounds concentrate in the permeate with an alcoholic content close to 40 mL/100 mL, and thus the retentate can be described as a “wine” free of alcohol and aroma (Labanda et al. 2009). The aroma compound concentrates can be treated with cyclodextrin polymers to remove volatile phenols before adding the aromatic fraction back (Liu et al. 2024; Sun et al. 2020). Another feasible method is using a membrane such as UF, which can retain molecules with molecular weights higher than 300 Da.

### 5.2 Enhancement of Sensory Attributes

Some phenolics lost during alcohol reduction methods might help with aroma release. For example, MF membranes can retain most phenolic compositions. However, the retention of gallic acid, catechin, and gallocatechin is lower than 10%, which can be attributed to the large pore diameter of the MF membrane allowing the diffusion of compositions with low molecular weights of 170 Da and 290 Da, respectively (Meija et al. 2019).

A previous study found that catechin can retain three red wine esters (ethyl octanoate, ethyl isobutyrate, ethyl butyrate) via hydrophobic interaction, resulting in decreased kiwi, strawberry, cheese, and fruity scents in wine (Liang et al. 2021). In the presence of catechin, the odor thresholds of ethyl octanoate and ethyl butyrate were increased by 3‐fold, suppressing the sensory perception of these volatile esters in wine. Consequently, the loss of catechin can sometimes enhance aroma release (Liang et al. 2021). Gallic acid (molecular weight 170 Da) and naringin (molecular weight 580 Da) were shown to retain ethyl benzoate, vanillin, and 2‐methylpyrazine in hydroalcoholic solution via an intricate combination of π–π stacking and hydrogen bonding (Liang et al. 2021). This interaction reduced the volatility of the aroma compositions and their relative perception, including warm, heavy, floral, fruity, sweet, creamy, vanilla, nutty, and cocoa‐like notes in the wine (Liang et al. 2021).

In addition to affecting aromas, membranes with good molecular weight cut‐off could preserve the good taste in wine (Sam et al. 2021). With the loss of low‐molecular‐weight phenolic compositions, the color, astringency, and bitterness of the wine will be altered (Pérez‐Magariño and González‐San José 2005). There are diverse small‐molecular‐weight phenolic compositions in red wines such as catechin, gallic acid et al., which play a significant role in determining red wines' astringency and bitterness.

### 5.3 Collection of Commercial Products

Except for the main usage of membrane separation, it can be inferred that membrane separation cannot only remove alcohol and its byproducts, such as grape spirit (alcohol separated by membrane) with low‐molecular‐weight phenolic composition, but also has commercial applications (Sun et al. 2020). For example, permeates containing enriched and purified low‐molecular‐weight polyphenols were proposed for food, pharmaceutical, or cosmetic industries (Garcia‐Castello et al. 2010). Or chromatographic techniques could be combined with membrane operations to isolate polyphenols in permeate in function of the desired product (Cassano et al. 2011).

## 6 Using Byproducts From Dealcoholized Wines to Compensate for Phenolic Loss

According to Section 5, it can be inferred that there are byproducts from dealcoholized wines, such as small‐molecular‐weight phenolic compounds, which can be enriched and obtained through chromatography. These small‐molecular‐weight phenolic compounds from red wines could potentially be used as food additives to compensate for the loss of similar compounds in other products and improve consumer acceptance as they prefer natural food additives as aforementioned. Alternatively, dealcoholized red wines that have had smoky taints removed could be added to other dealcoholized red wines that have experienced phenolic loss. This process could improve wine complexity and overall wine quality (Wang and Spence 2019).

## 7 Conclusions

The growing demand for low‐alcohol and nonalcoholic wines has led to significant research and development in alcohol reduction methods. This review has examined various techniques for reducing alcohol content in red wines, focusing on their impact on phenolic compositions, which are crucial for wine quality and health benefits. Based on these techniques, it can be inferred that supercritical CO_2_ extraction could result in some phenolic loss. While the operation cost may be high, the removed alcohol can serve as a byproduct, increasing industrial utility. Meanwhile, spinning cone column and membrane separation techniques can remove both smoky taints and alcohol content simultaneously. However, currently researchers have mainly used membrane separation to remove alcohol from red wines, which can lead to a significant loss of small‐molecular‐weight phenolic compositions, potentially resulting in unstable sensory attributes in red wines.

Therefore, there is a need to establish a comprehensive database on the sensory attributes of dealcoholized red wines produced by diverse methods. This database would offer multiple benefits including: knowledge consolidation by standardizing currently fragmented sensory data across studies; decision support for producers facing financial risks when investing in dealcoholization equipment; alignment of consumer preferences with specific sensory profiles to better target market segments; and quality improvement by identifying common sensory defects associated with specific methods to guide targeted innovation. Meanwhile, machine learning techniques are being developed to help researchers select the most appropriate methods that minimize negative impacts on dealcoholized red wines. Moreover, as there are flaws in the sensory attributes of heavily dealcoholized wines (those with alcohol content reduced by more than 2% v/v), it remains to be explored whether feasible methods—such as ultrasound or pulsed electric fields—can be combined with existing dealcoholization techniques to form integrated food processing approaches that address these issues. These combined techniques may also enhance the efficiency of the dealcoholization process.

## Author Contributions

Conceptualization, methodology, and writing – original draft, J.A.; Data acquisition, Z.Z., A.J., and M.T.; Funding acquisition and project administration, S.J.; Project administration, writing – review and editing, Y.L. All authors have read and agreed to the published version of the manuscript.

## Ethics Statement

The authors have nothing to report.

## Consent

The authors have nothing to report.

## Conflicts of Interest

The authors declare no conflicts of interest.

## Supporting information


**Table S1.** The phenolic composition in red wines
**Table S2.** Smoky phenols in red wines (Wang & Chambers IV, 2018)

## Data Availability

The original contributions presented in the study are included in the article; further inquiries can be directed to the corresponding authors.
