# Supplementary material for: A Critical Review of Alcohol Reduction Methods for Red Wines From the Perspective of Phenolic Compositions
Source: Food Sci Nutr. 2025 Jun 8;13(6):e70401. doi: 10.1002/fsn3.70401 (PMC12146494; doi:10.1002/fsn3.70401)
Supplement: Supplementary file 1 — Table S1. The phenolic composition in red wines Table S2. Smoky phenols in red wines (Wang & Chambers IV, 2018) [file FSN3-13-e70401-s001.docx]

Supplementary Table 1. The phenolic composition in red wines

| Phenolic type | Name | Polar (glucoside) | Molecular weight |
| --- | --- | --- | --- |
| Flavanols (seed or skins) | Catechin | No | 290 |
|  | Epicatechin | No | 290 |
|  | Epicatechin-gallate | 1 | 442 |
|  | Epigallocatechin | 1 | 458 |
|  | Gallocatechin | No | 306 |
| Anthocyanins (accumulate in the skin as glucosides) | Delphinidin-3-O-glucoside | Yes | 465 |
|  | Cyanidin 3-O-glucoside | Yes | 449 |
|  | Petunudin 3-O-glucoside | Yes | 479 |
|  | Malvidin-3-O-glucoside | Yes | 493 |
|  | Delphinidin3-O-acetylglucoside | Yes | 507 |
|  | Petunidin 3-O-acetylglucoside | Yes | 521 |
|  | Malvidin 3-O-acetylglucoside | Yes | 535 |
|  | Petunidin 3-O-ρ-coumaroylglucoside | Yes | 625 |
|  | Peonidin 3-O-ρ-coumaroylglucoside | Yes | 609 |
|  | Malvidin-3-O-ρ-coumaroylglucoside | Yes | 639 |
| Flavonols | Quercetin | No | 302 |
|  | Quercetin-3-glucoside | Yes | 464 |
|  | Kaempferol | No | 286 |
| Flavanone | Astilbin | Yes | 450 |
| Phenolic acid | Caffeic acid | No | 180 |
|  | Gallic acid | No | 170 |
|  | ρ-coumaric acid | No | 164 |
|  | Protocatehuic acid | No | 154 |
|  | Resveratrol | No | 228 |
|  | Caftaric acid | No | 312 |
|  | Syringic acid | No | 198 |
|  | Tyrosol | No | 138 |

Supplementary Table 2. Smoky phenols in red wines (Wang & Chambers IV, 2018)

| Name | Polar (glucoside) | Molecular weight |
| --- | --- | --- |
| o-cresol | No | 108 |
| m-cresol | No | 108 |
| ρ-cresol | No | 108 |
| thymol | No | 150 |
| 2,5-dimethylphenol | No | 122 |
| 3,4-dimethylphenol | No | 122 |
| 2,4-dimethylphenol | No | 122 |
| 2,4,6-trimethylphenol | No | 136 |
| 3-ethyl-5-methylphenol | No | 136 |
| carvacrol | No | 150 |
| guaiacol | No | 124 |
| 4-ethylguaiacol | No | 152 |
| 2-methoxy-4-methylphenol | No | 138 |
| 2-methoxy-4-vinylphenol | No | 150 |
| eugenol | No | 164 |
| isoeugenol | No | 164 |
| 2,6-dimethoxylphenol | No | 154 |
| 4-allyl-2,6-dimethoxyphenol | No | 194 |

Wang, H., & Chambers IV, E. (2018). Sensory characteristics of various concentrations of phenolic compounds potentially associated with smoked aroma in foods. *Molecules*, *23*(4), 780.
